# A discrete choice experiment to explore patients’ willingness to risk disease relapse from treatment withdrawal in psoriatic arthritis

**DOI:** 10.1007/s10067-016-3452-1

**Published:** 2016-10-31

**Authors:** Claire Rothery, Laura Bojke, Gerry Richardson, Chris Bojke, Anna Moverley, Laura Coates, Liz Thorp, Robin Waxman, Philip Helliwell

**Affiliations:** 1 Centre for Health Economics, University of York, York, YO10 5DD UK; 2 Leeds Institute of Health Sciences, University of Leeds, Leeds, LS2 9JT UK; 3 Leeds Institute of Rheumatic and Musculoskeletal Medicine, and Leeds Musculoskeletal Biomedical Research Unit, Leeds Teaching Hospitals Trust, University of Leeds, Leeds, UK; 4 Bradford Institute for Health Research, Bradford, UK

**Keywords:** Discrete choice experiment, Patient preferences, Psoriatic arthritis, Relapse risk

## Abstract

The objective of this study is to assess patient preferences for treatment-related benefits and risk of disease relapse in the management of low disease states of psoriatic arthritis (PsA). Focus groups with patients and a literature review were undertaken to determine the characteristics of treatment and symptoms of PsA important to patients. Patient preferences were assessed using a discrete choice experiment which compared hypothetical treatment profiles of the risk and benefits of treatment withdrawal. The risk outcome included increased risk of disease relapse, while benefit outcomes included reduced sickness/nausea from medication and changes in health-related quality of life. Each patient completed 12 choice sets comparing treatment profiles. Preference weights were estimated using a logic regression model, and the maximum acceptable risk in disease relapse for a given improvement in benefit outcomes was elicited. Final sample included 136 patients. Respondents attached the greatest importance to eliminating severe side effects of sickness/nausea and the least importance to a change in risk of relapse. Respondents were willing to accept an increase in the risk of relapse of 32.6 % in order to eliminate the side effects of sickness/nausea. For improvements in health status, the maximum acceptable risk in relapse was comparable to a movement from some to no sickness/nausea. The study suggests that patients in low disease states of PsA are willing to accept greater risks of relapse for improvements in side effects of sickness/nausea and overall health status, with the most important benefit attribute being the elimination of severe sickness or nausea.

## Introduction

Psoriatic arthritis (PsA) is an inflammatory arthritis affecting the joints and connective tissue and is associated with psoriasis of the skin or nails [1]. It is characterised by pain, swelling and inflammation of the joints. Psoriasis affects 2–3 % of the UK population, and the prevalence of inflammatory arthritis in patients with psoriasis is estimated to be up to 30 % [2, 3].

There is currently no cure for PsA and conventional disease-modifying anti-rheumatic drugs (DMARDs) have shown limited efficacy in clinical trials [4]. Modest efficacy has been shown for sulfasalazine [5] and leflunomide [6] with conflicting evidence shown for methotrexate (MTX) [7]. However, the use of anti-tumour necrosis factor (TNF) therapy for the treatment of inflammatory arthritis has revolutionised therapeutic options in PsA. TNF inhibitors are highly effective against both skin and joints but they are expensive and associated with potentially serious adverse events. With the introduction of these agents, remission in PsA is now an achievable target. However, in some clinical situations, the risks and side effects associated with treatment may outweigh the benefits of therapy [8]. The effects of long-term immunosuppressant therapy are unknown. The economic impact of psoriasis is also important [9–11]. If patients experience some degree of treatment interruption while remaining in remission, this would significantly reduce the treatment costs for PsA patients.

It has recently been shown that remission in PsA may be sustained despite treatment interruption [12]. However, two further studies have suggested that complete withdrawal of treatment leads to relapse in the majority of patients [13, 14]. In order for patients to make an informed decision about whether or not to have their treatments withdrawn or scaled down, they must be advised of both the benefits (e.g. fewer side effects associated with treatment) and risks (e.g. risk of relapsing after a state of remission). Patient-preference methods such as discrete choice experiments (DCEs) [15, 16] have increasingly been used to quantify the relative importance of the benefits and risks of treatment to patients [17, 18]. The primary objective of this study was to undertake a DCE to quantify the trade-off between benefit and risk preferences for patients in low disease states of PsA in order to inform the non-inferiority margin in risk of relapse between staying on treatment and withdrawing from treatment. This can be achieved by asking individuals to state preferences over particular features of treatment. Specifically, the objective was to estimate the trade-off between the primary outcome measure of risk of relapse (flare of disease), side effects of treatment and symptoms of PsA. The maximum acceptable risk in negative outcomes that patients are willing to accept for a given improvement in benefit outcomes represents the level of non-inferiority from the patients’ perspective. If a future randomised control trial (RCT) were to be planned based on patient preferences, this elicited non-inferiority margin could be used in a standard sample size and power calculation to determine the sample size required for a full non-inferiority RCT.

## Methods

### Review of literature

A literature review was conducted to determine the set of characteristics or attributes (e.g. symptoms of PsA, side effects of treatment) important to patients. However, in the initial stages of reviewing the literature, it became clear that ‘traditional’ systematic searching methods using keywords was not possible. This was because identifying the key terms and Medical Subject Headings (MeSH) that capture the range of relevant literature is difficult without having pre-existing knowledge of the important characteristics or attributes to be determined. Therefore, a ‘pearl growing’ technique was used to identify the relevant literature. This approach uses a pool of ‘initial pearls’ identified by experts in the field of rheumatology and experienced in the treatment of PsA (PH, LC, AC) to grow the literature through references and/or citations until all relevant studies have been identified. Repetition of the pearl growing process was continued up to the point that it was felt that no additional information from the literature could be obtained. Details on symptoms of PsA and treatment characteristics, particularly adverse effects, were extracted from these ‘pearls’. A hand-searching approach was used to extract the themes from these studies. We first identified a provisional list based on the first study examined and then subsequently updated the list as further studies from the identified sample were reviewed. The Summary of Product Characteristics (SPC) was used as an additional source of information for obtaining the adverse effects associated with medications for PsA.

Twenty-one studies were initially identified. From these, a further 464 studies were identified through reference searching, of which 343 were excluded as not relevant to the topic. Titles and abstracts of the remaining 142 studies were screened for inclusion. Of these, full copies of 74 studies were retrieved and reviewed in order to identify the characteristics of treatment important to patients. None of the studies were specifically designed to examine the attributes of treatment. Most of the studies considered outcome or disease activity measures, efficacy and safety of treatments, burden of disease, benefits of therapy and/or potential factors to predict remission and sustained minimal disease activity. However, within these studies, a number of important characteristics were identified: (i) ability to work and time lost from work; (ii) ability to undertake household tasks and activities at home; (iii) participation in social activities; (iv) patient time spent caring for PsA; (v) frequency of hospital inpatient stays, outpatient appointments and out-of-pocket expenses; and (vi) anxiety, stress, depression, pain, fatigue and sleep disturbance. The major adverse effects associated with treatment were identified as follows: (i) nausea, sickness, fatigue, headache, diarrhoea; (ii) infections and allergic reactions; (iii) abdominal and musculoskeletal pain; (iv) hair loss; (v) need for regular blood tests; and (vi) implications for pregnancy.

### Focus groups

Focus groups with PsA patients were used to refine and expand upon the treatment characteristics revealed by the literature into a set of attributes and levels to be used in the design of the DCE. The focus groups encouraged patients to discuss the symptoms of PsA, the side effects of medication and the potential consequences of stopping treatment (in particular, the risk of relapse). They were also used to provide a guide to the range of relapse rates that patients might be willing to accept. Patients with a wide spectrum of disease characteristics presenting for routine rheumatology appointments at St Luke’s Hospital, Bradford UK participated in two separate focus groups of 12 and 6 participants; the discussions were structured by an expert in qualitative analysis/focus groups and each session lasted approximately 1 h 30 min. Patient consent was obtained according to the Declaration of Helsinki and the study was approved by the National Health Service (NHS) Research Ethics Committee (REC) in UK.

Participants discussed how PsA affects their usual activities, work and ability to interact with friends and family due to pain, fatigue and swelling. They also discussed how medication eases the symptoms of PsA and generally allows them to lead a fairly normal life. Flares of disease happen quite often but less so on medication. Some participants expressed concern about the long-term consequences of taking medication. Side effects while on medication were particularly noted for methotrexate, which can cause nausea, vomiting and diarrhoea. The majority of participants did not want to come off their current medication because of the risk of relapse and the time required for medication to become effective again after a break. Participants indicated that the side effects of treatment were not sufficiently bad to consider treatment withdrawal. However, some participants would consider the possibility of treatment withdrawal due to concerns about the long-term consequences of treatment and possible toxicities. Some participants indicated that they had stopped medication previously and noted that it was good not to experience the side effects of treatment; however, symptoms of PsA generally returned later.

### Discrete choice experiment

To inform the feasibility of a future trial of treatment withdrawal, the willingness of patients to withdraw from treatment needs to be determined. A DCE was used to quantify the trade-off between benefits of medication and risk of relapse for patients in low disease states of PsA. The attributes in the DCE were used to represent the characteristics of treatment and symptoms of PsA important to patients, while the levels represent the values that these attributes may take. The attributes and levels identified by the literature review were supplemented by the focus group information using a thematic approach. This was done by group consensus and with input from a patient representative. The final set of attributes and levels were chosen to minimise the number of possible levels (for cognitive as well as computational ease) and reduce any overlap between attributes.

The dimensions of the EQ-5D [19, 20] were found to fit the symptoms of PsA very well. These were used to represent five of the attributes: mobility, self-care, usual activities, pain or discomfort and anxiety or depression. The use of the EQ-5D dimensions to represent the symptoms of PsA has the added advantage that health-related quality of life utility values are available (i.e. values that reflect an individual’s preference for different health outcomes, which are measured on an interval scale with zero reflecting states of health equivalent to death and one reflecting perfect health). A further two attributes were added, one to represent the risk of relapse (most often described as a flare) and one to represent the side effects of treatment, specifically sickness and nausea. The range of levels for each attribute were chosen to represent the boundaries of PsA health and the maximum range over which respondents would be willing to accept trade-offs among attributes.

Once attributes and levels were determined, they were combined into treatment profiles that describe hypothetical scenarios. In defining the choice sets, consideration was given to the cognitive burden on respondents; for example, a full factorial design representing the full range of combinations of seven attributes with three levels each would result in 3^7^ = 2187 possible health outcome profiles. To reduce the cognitive burden, the clinical team (AC, LC, PH) selected the attributes most relevant to patients who would be considered for treatment withdrawal in PsA, i.e. patients in stable low disease activity. Three health states were selected from the EQ-5D: (i) Level 11111 with utility 1.0 (i.e. perfect health); (ii) Level 11221 with utility 0.760; and (iii) Level 11222 with utility 0.689; representing a clear ranking in severity of symptoms, i.e. (i) is better than (ii), which in turn is better than (iii) in terms of health-related quality of life. Table 1 shows the final set of attributes and levels used in the experimental design of the DCE.

**Table 1:**
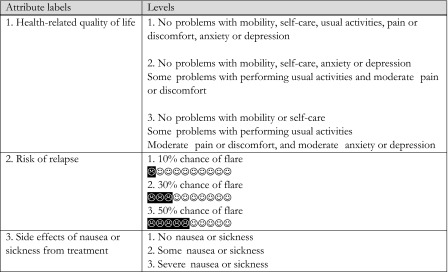
Attributes and levels used in the DCE

In a DCE, every respondent makes a discrete choice between alternative A or alternative B, where each alternative represents a bundle of attributes at different levels. In each choice, the rational respondent will choose the option that yields the highest level of utility or benefit, i.e. an individual will choose A over B if U_A_(C_A_, S) > U_B_(C_B_, S), where U(.) is the utility derived from the choice, C_A_ and C_B_ are the combination of attributes and levels associated with option A and B, respectively, and S represents the characteristics of the individual that influences their preference. A fractional factorial design for the DCE was used to estimate the trade-offs as efficiently as possible [15]. The best experimental design based on 12 choice sets was derived (by ensuring level balance, orthogonality, minimal overlap and utility balance in the choice sets [21–24]. In order to check for ‘rational’ responses, the design also included three choice sets with a dominated answer, i.e. one of the pairwise options had worse levels in all attributes than the alternative choice. A warm-up choice set, using the same format as the actual choice sets, and introductory text explaining the objectives of the survey were included. The questionnaire was piloted with the patient representative on the project and two patients attending clinic. The purpose of the pilot was to establish whether the respondents understood the choice sets, whether the attributes were traded, whether one attribute dominated the others and whether the responses were internally consistent. The final version of the survey was sent for ethics approval and mailed to 644 PsA patients considered to have minimal disease activity (as defined by Coates et al. 2010 [21]) on the Bradford Psoriatic Arthritis Disease Register. A copy of the DCE is presented in the Supplementary Appendix.

### Statistical analysis

The patterns of respondent choice was analysed using a conditional logic regression model. In this model, the dependent variable was the choice between A and B and the explanatory variables were the levels of the attributes, which measure the marginal utility of changes in the characteristics, i.e. differences between the weights (coefficients of the regression model) for the explanatory variables indicate the relative importance of movements between levels of each attribute. The marginal rate of substitution or the trade-off between attributes and levels was estimated by dividing one coefficient by another. This was used to estimate the maximum acceptable risk (MAR) in relapse that respondents were willing to accept (i.e. the mean level of risk in negative outcomes) for a given improvement in benefit outcomes. It was calculated as the change in risk of relapse that would exactly offset the perceived benefit of an improvement in sickness/nausea or health status. Therefore, the MAR represents the level of non-inferiority in the risk of relapse from the patients’ perspective.

## Results

### Patient sample characteristics

Of the 644 patients who were sent the questionnaire, 247 (38 %) completed and returned it. Table 2 shows the characteristics of respondents who participated in the DCE. Gender balance was similar across participants, who were of an average age of 55 years and with PsA for an average of 8.6 years. The majority of participants considered their PsA to be controlled (64.8 %), and most were receiving treatment with either methotrexate alone or in combination with other DMARDs or biologics (58.7 %). A sizeable proportion of participants (23.9 %) rated their own health as severely impaired with an EQ-5D utility value of less than 0.5, where a value of 1.0 represents full health.

**Table 2:** Characteristics of respondents participating in the DCE

Patient characteristics	Number of patients (% of total)
Gender	
Male Female	115 (46.6 %) 132 (53.4 %)
Age (average age, 55 years)	
<55 years old ≥55 years old	107 (43.3 %) 139 (56.3 %)
PsA controlled (from the patients’ perspective)	
Yes No Missing	160 (64.8 %) 76 (30.8 %) 11 (4.4 %)
Duration of PsA (average 8.6 years)	
<9 years ≥9 years Missing	141 (57.0 %) 78 (31.6 %) 28 (11.4 %)
Medication	
Methotrexate alone Other DMARDs alone Biologics alone Methotrexate in combination with other DMARDs or biologics Other DMARDs in combination with biologics (no methotrexate) No medication	77 (31.2 %) 38 (15.4 %) 30 (12.2 %) 68 (27.5 %) 8 (3.2 %) 26 (10.5 %)
EQ-5D utility	
<0.000 0.000– <0.500 0.500– <0.750 0.750–1.000	31 (12.6 %) 28 (11.3 %) 106 (42.9 %) 82 (33.2 %)

An unexpectedly large proportion of the responses to the DCE (45 %) gave an ‘irrational’ answer to at least one of the three choice sets which had a dominated answer, i.e. the respondent did not choose the option that clearly yielded the highest level of benefit. The reasons for this are unclear; these participants may not have been paying close attention to the alternative choice sets, may have misunderstood the nature of the exercise or may have ‘perverse’ preferences. Consequently, their responses were excluded from the sample.^1^ The final participant sample size which answered all three choice sets with a dominated answer correctly was 136.

### Importance weights

**Fig. 1:**
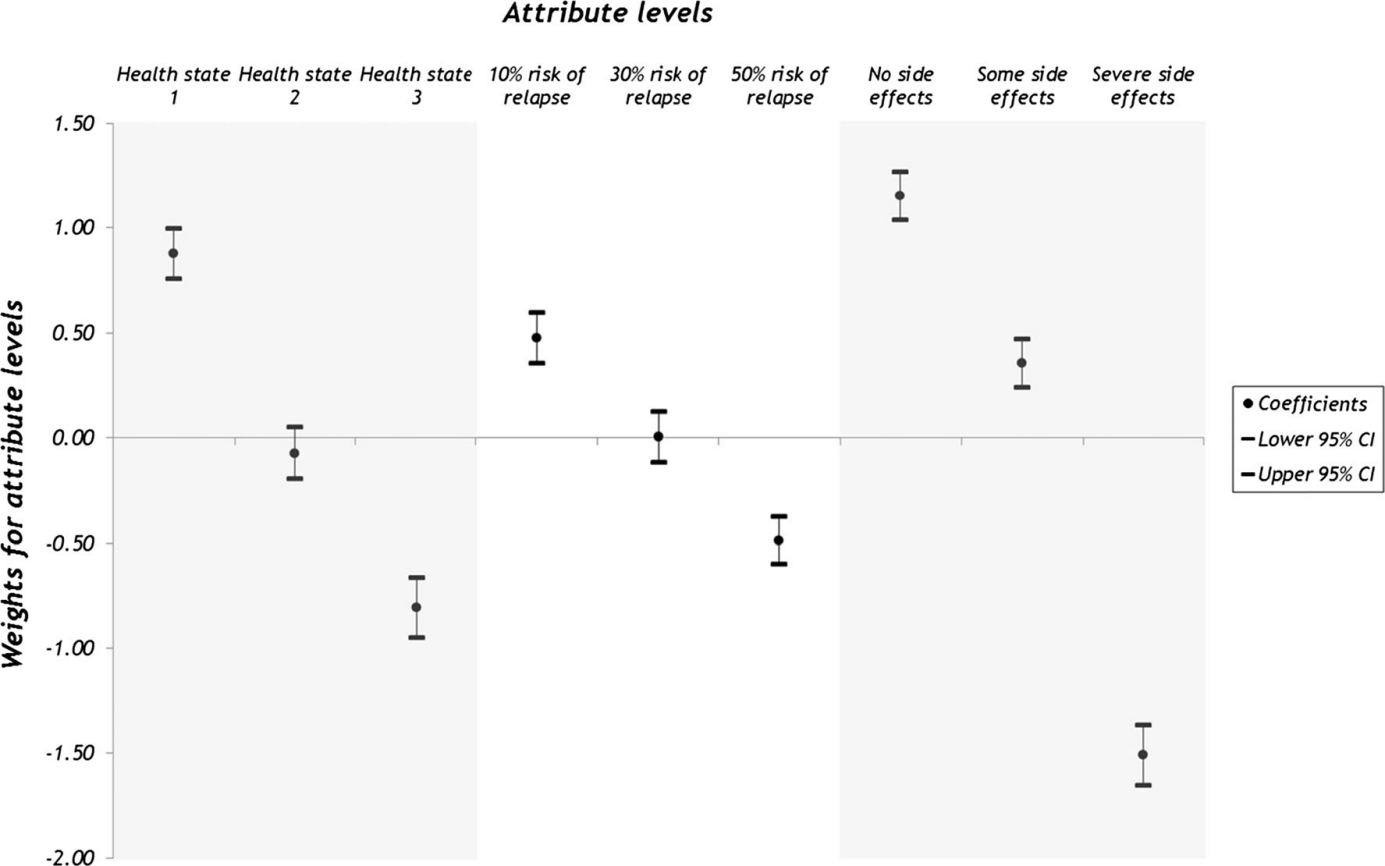
Weights for the attribute levels

Figure 1 shows the estimated weights and 95 % confidence intervals (CIs) for the attributes and levels. Differences between adjacent weights for attribute levels indicate the relative importance of moving from one level to an adjacent level of the attribute, i.e. the greater the difference the more important the change from one level to the next. Where the confidence intervals do not overlap for adjacent levels in a particular attribute, the mean estimates are statistically different from each other at the 5 % level of significance. Figure 1 indicates that respondents attached greater importance, by an order of magnitude of double, of moving from severe side effects of nausea and sickness to some nausea/sickness (weight, 1.8668) than to moving from some to no nausea/sickness (weight, 0.7996). The relative importance of moving from a relapse risk of 50 % to a reduced risk of 30 % (weight, 0.4906) was similar to the movement from 30 to 10 % risk of relapse (weight, 0.4726). For health status, slightly more importance was attached to the movement from health state 2 with ‘some problems with usual activities, moderate pain and discomfort’ to health state 1 with no problems in these dimensions (weight, 0.9463) than the movement from health state 3, which additionally had ‘moderate anxiety and depression’ to health state 2 (weight, 0.7373). None of the confidence intervals for the adjacent levels of any attribute overlapped suggesting that the levels were statistically different from one another.

**Fig. 2:**
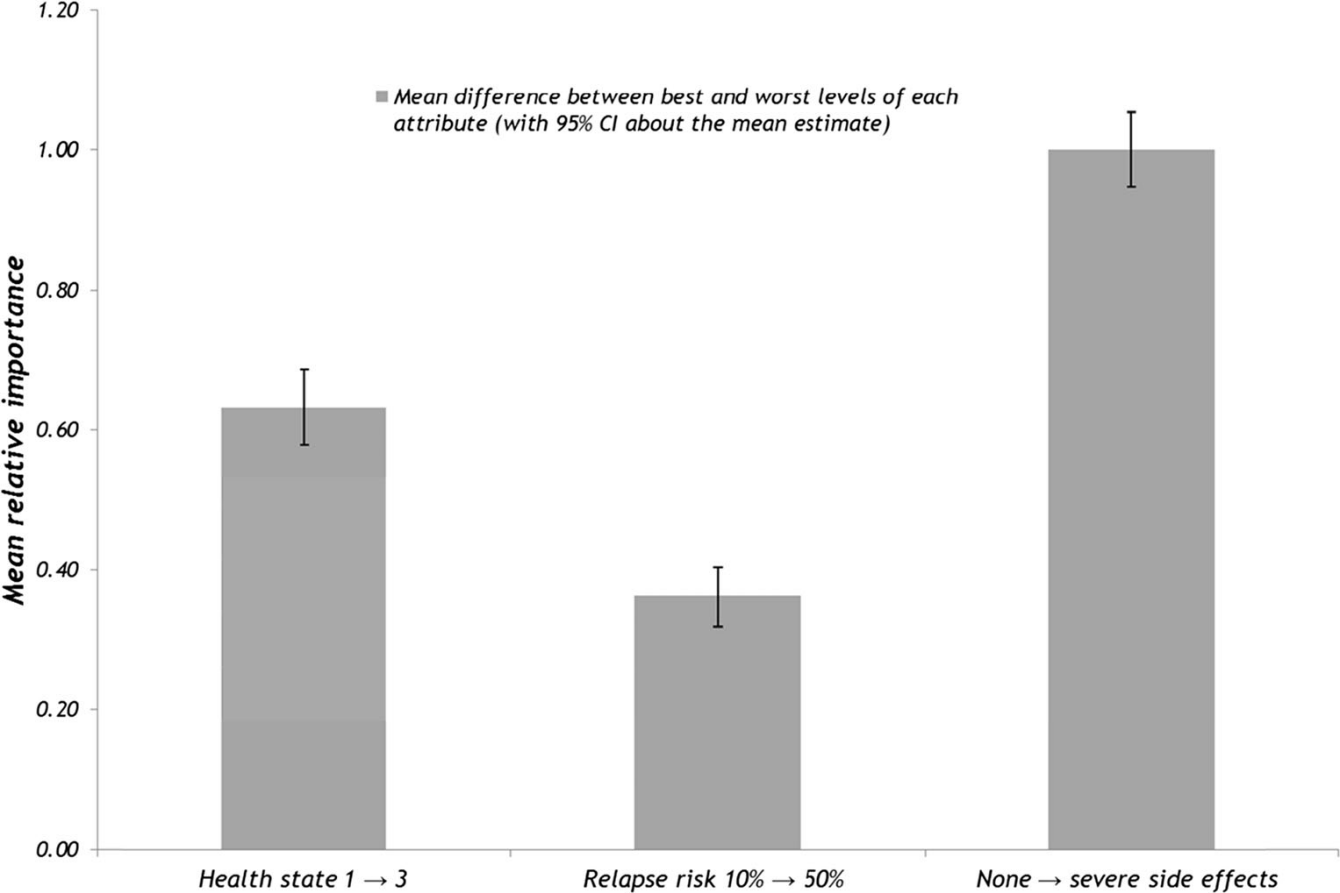
Relative importance of the attributes

The difference between adjacent weights of one attribute can be compared with the difference between adjacent weights of a different attribute to understand how comparable the magnitude of the change is across attributes. For example, the distance between weights for the best and worst levels of an attribute can indicate the relative importance of one attribute to any other attribute. Figure 2 shows the relative importance of the attributes scaled such that the most important attribute had a mean importance score of 1.0. It indicates that respondents attached the greatest importance to eliminating severe side effects of nausea and sickness and the least importance to the change in relapse risk.

### Trade-off between attributes and levels

Table 3 presents the maximum acceptable level of risk of relapse that respondents were willing to accept in exchange for improvements in levels of sickness/ nausea and health status. The MAR was based on the importance of reducing the relapse risk from 50 to 30 %. For example, the difference in the weight between the levels of 50 and 30 % risk of relapse in Fig. 1 is 0.4906. Therefore, each percentage point increase in risk decreases utility by 0.4906/(50−30 %) = 0.0245. The difference in weight between the levels of the other attributes was then measured on the same scale in order to determine the relative trade-off between attributes. For example, the difference in the weight between ‘some’ and ‘no’ sickness/nausea in Fig. 1 is 0.7997, which equates to 0.7997/0.0245 = 32.6 % increase in the risk of relapse, i.e. respondents, on average, were willing to accept an increase in the risk of relapse of 32.6 % to eliminate the side effects of some sickness/nausea. Therefore, the MAR in Table 3 represents the change in risk of relapse which exactly offsets the perceived benefit of improvements in levels of sickness/nausea and health status. Respondents were willing to accept a very large increase in risk (76.1 %) in exchange for an improvement in ‘severe’ to ‘some’ sickness/nausea. For improvements in health status (health state 3 → 2 and 2 → 1), the MAR was comparable to each other and to ‘some to no’ sickness/nausea.

**Table 3:** Maximum acceptable risk in relapse that respondents were willing to accept for improvements in levels of sickness/nausea and health status

Improvements in perceived benefits	Maximum acceptable risk
Improvements in side effects	
Severe to some sickness/nausea Some to no sickness/nausea	76.1 % 32.6 %
Improvements in health status	
Health state 3 to health state 2 Health state 2 to health state 1	30.1 % 38.6 %

Based on the importance of reducing the relapse risk from 50 to 30 %. Each percentage point increase in risk decreases patients’ utility by 0.4906/(50−30) = 0.0245

## Discussion

This study is one of the first to quantitatively assess the factors that influence a patient’s decision to withdraw from medication when in a low disease state of PsA. The focus group discussions highlighted the concerns of participants about the long-term consequences and side effects associated with medication use and also the risk of relapse (‘flares’) if treatment were withdrawn. The DCE was used to quantify the trade-off between benefit and risk preferences for patients in order to establish the non-inferiority margin where increased risk in negative outcomes offset perceived benefits.

The study has two main findings. Firstly, the most important benefit attribute was the elimination of severe side effects of sickness and nausea. This was ranked more important than moving from a health state with moderate pain or discomfort, moderate anxiety or depression and some problems with performing usual activities (no problems with mobility and self-care) to a health state with none of these problems. Secondly, the results suggest that patients are willing to accept a very large increase (over 30 %) in risk of relapse in exchange for improvements in levels of sickness/nausea and health status. These findings suggest that an assessment of the benefits and risks of treatment for PsA is an important consideration for making an informed choice about treatment withdrawal. Patient preferences play a key role and future clinical trials should consider patients’ current symptoms and their willingness to accept different levels of relapse risk. To this end, the elicited non-inferiority margin from the patient’s perspective (along with an estimate of variability) could be used in a standard sample size and power calculation to determine the sample size needed for a full non-inferiority randomised controlled trial (RCT). For example, Table 3 indicates that 32.6 % is the non-inferiority margin in relapse rate between staying on treatment and withdrawing from treatment that patients are willing to accept in compensation for elimination of some side effects of sickness/nausea. In this case, the design of the non-inferiority RCT could be based on the hypothesis that treatment withdrawal is inferior to continuation of therapy by the minimum important difference of 32.6 % in the primary outcome of risk of relapse (in exchange for elimination of some sickness/nausea). This is analogous to designing a trial with a sample size based on what the investigator believes to be a clinically important difference in outcomes; the only difference here is that the level of non-inferiority is from the patients’ perspective based on their view of the relative importance of the benefits and risks used in the assessment. However, an important caveat is that the design of an RCT should not be totally driven by a limited number of trade-offs (for example, in this case the trade-off is limited to the elimination of some sickness/nausea, which was considered important to patients at the focus groups). Instead, the DCE should be used to help guide the design of an RCT by providing a better understanding of patient preferences and the trade-offs between attributes that individuals are willing to accept.

DCEs have been used widely in many areas of healthcare to elicit patient preferences, but they have important limitations. One limitation is that patients evaluate hypothetical choice sets, which are intended to simulate real decisions; however, the responses may not accurately reflect real behaviours as respondents do not experience the consequences of their decisions. Therefore, differences can arise between stated and actual preferences. Another important limitation is the number of attributes that can be included. For ease of cognitive burden and practical feasibility, DCEs can only consider a limited number of attributes and levels. In this respect, decisions about the most important attributes to include in the DCE are inevitable. In order to quantify the trade-off in risk of relapse relative to the other attributes, we assumed that risks were linear between the two levels of relapse (30 and 50 %) but risk preferences between these levels may not be linear in reality.

This study had a number of other important limitations. The response rate to the postal questionnaire was quite low at 38 %. More substantial, however, was the unexpectedly large proportion of the responses (45 %) which gave an irrational answer to at least one of the three choice sets with a dominated option. Given that we cannot be clear of the reason for the irrational choices, the final sample only included participants who correctly answered all three choice sets, which resulted in quite a small sample size of 136. Moreover, it was not possible to explore how different medications and patient characteristics might affect patient preferences as any split in the sample into subgroups would lead to very small sample sizes for each group. The results reflect a survey population who were mostly well in terms of arthritis (so may accept risk of pain/flare) and had chronic disease with long duration. The outcomes of such a survey may be different in patients with early disease when first on therapy. We are not aware of any other DCEs in PsA which have examined the risk of relapse that patients are willing to accept for other characteristics. There have been several studies focused on patient preferences in rheumatoid arthritis [22–26]. A recent study by Harrison et al. (2015) showed societal preference values for rheumatoid arthritis [27]. In this study, participants were willing to trade a year of life expectancy over a 10-year period to increase the probability of benefiting from treatment, and two-thirds of a year to reduce minor or serious side effects to the lowest level [27]. In conclusion, this study provides insight into the benefit and risk preferences of patients who may be considered for withdrawal of treatment in PsA but is limited by the size of the sample. Notwithstanding this, the study suggests that patients in low disease states of PsA are willing to accept greater risks of relapse for improvements in side effects of sickness or nausea and overall health status, with the most important benefit attribute being the elimination of severe sickness or nausea.

## Footnotes

1 A sensitivity analysis was conducted on the full sample of 247 respondents. The results were very similar for movements between the levels of side effects of sickness/nausea and quite similar for relapse risk. However, the results were very different for movements between levels of health status. Given the irrational nature of the additional 111 responses, it is difficult to interpret this finding.
